# Advances in the Current Understanding of the Role of Extracellular Vesicles in Allergy, Autoimmunity and Immune Regulation

**DOI:** 10.3390/ijms232214311

**Published:** 2022-11-18

**Authors:** Krzysztof Bryniarski, Katarzyna Nazimek

**Affiliations:** Department of Immunology, Jagiellonian University Medical College, 18 Czysta St., 31-121 Krakow, Poland

Cells release extracellular vesicles (EVs), such as exosomes and microvesicles, both under physiological and pathological conditions, making EV-dependent signaling cascades a very precise system of intercellular communication. Over the last decade, the number of articles published annually on research on exosomes has increased more than tenfold. However, despite such rapid scientific progress, our current level of understanding of the biological roles of EVs is still at the beginning of a complicated path to fully applying this knowledge in practice. Moreover, the transfer of genetically encoded information via EVs is unprecedented and breaks the paradigm.

The involvement of EVs in systemic intercellular communication and their capability of delivering various immune regulatory molecules, including non-coding RNAs, such as microRNAs (miRNAs), made them a promising candidate for diagnostic and therapeutic applications in various immune-related disorders. Accordingly, the increasing prevalence of allergic and autoimmune diseases urges researchers and clinicians to search for new and efficient treatments with reduced toxic side effects and increased specificity to inhibit the detrimental immune responses underlying these diseases. All of these aspects seem to be achievable with EV-based therapies.

Therefore, the Special Issue “Extracellular Vesicles in Allergy, Autoimmunity and Immune Regulation 2.0” was conceived to present the current knowledge on the functions of EVs in allergies and autoimmune diseases, as well as in the regulation of immune responses, which would greatly support the process of uncovering the immunotherapeutic potential of EVs.

An interesting study by Hudec et al. [1] investigated the interaction of celiac patient-derived dendritic cells with T cells, with a special focus on possible HLA-DQ transfer via EVs. Monocyte-derived dendritic cells from patients suffering from this autoimmune disease were demonstrated to potently activate T lymphocytes from healthy subjects in an HLA-DQ-dependent manner. A similar effect was achieved by treating T cells with conditioned growth medium from a culture of monocyte-derived dendritic cells, and transferred HLA-DQ molecules co-localized with CD63 within recipient T lymphocytes. Thus, the authors concluded that HLA-DQ could be released by dendritic cells in EVs to potentiate the autoimmune reactivity of T lymphocytes and possibly to diminish the induction of regulatory T cells. These observations are a great addition to the current understanding of the pathogenesis of celiac disease.

Another research article by Gómez-Chávez et al. [2] focused on the role of microbial EVs in the modulation of autoimmune response in the mouse model of imiquimod-induced psoriasis. The authors showed that EVs secreted by a commensal strain of *Staphylococcus epidermidis* decrease the production of proinflammatory cytokines by HaCaT keratinocytes as well as alleviate the cutaneous inflammation in psoriatic mice. A downstream proteomic analysis of staphylococcal EVs suggested that the anti-inflammatory effect is induced by the contained bacterial enzymes (including arginine deiminase, catalase, and superoxide dismutase), possibly acting in an IL-36Ra-dependent manner. These findings reveal the important role of EVs from commensal microbiota in the modulation of autoimmune responses.

The review article by Matsuzaka and Yashiro [3] summarizes recent advances in scientists’ understanding of the mechanisms of self-tolerance and its breaking that leads to autoimmune response development, with a special emphasis on the functions of EVs and their miRNA cargo. It was highlighted that uncovering the mechanism of EVs’ secretion and the final result caused by its enhancement or inhibition is one of the crucial steps to clarifying the action of EVs in particular conditions. This would greatly support the application of EVs and EV-transmitted miRNAs to cell-free drug delivery systems. In addition, innovative thinking led the authors to conclude that removing a harmful subpopulation of EVs from the patient’s circulation could be considered an effective treatment for a given disease.

From a clinical and therapeutic point of view, introducing EVs into routine practice is still fraught with many challenges. The review article by Philip Askenase [4] discusses the contribution of “carrier effects” to the final biological effect induced by EVs and the transmitted cargo. The main advantages of EV-dependent “carrier effects” are related to the biocompatibility, the resistance to harsh conditions, and the fact that molecules encapsulated in EVs are delivered to the target cell at much higher concentrations than those that freely circulate in the blood. The latter property is further supported by the highly specific and selective targeting of cells provided by EVs, which together make it possible to achieve an enhanced biological effect at a low dose of immune regulatory molecule (e.g., miRNA).

Another key challenge is finding the most effective route of EV administration that is acceptable to the patient. Along these lines, the review article by Cieślik et al. [5] discusses the advantages and current limitations of the possibility of administering EVs by the oral route. While the most difficult issues to achieve are the dosing protocol and the optimal absorption of EVs in the gastrointestinal tract, the primary benefits of this approach include very low invasiveness and a good feasibility level. It is also worth noting that EVs of various sources, including edible plants, dairy products, commensal microbiota, and mammal cells, could likely be uptaken by intestinal cells to induce the expected immune regulatory effects locally and in distant tissues, and this phenomenon is now extensively studied.

The goal of this Special Issue was to comprehensively discuss the current knowledge on the immune regulatory potential of EVs in allergies and autoimmunity as well as to outline the future perspectives for their therapeutic applications. Notably, EVs’ biology and clinical applicability are tremendously complex study areas that require open-minded research approaches and innovative solutions to achieve the intended goals.
